# Supplementary material for: Sedimentation of large, soluble proteins up to 140 kDa for 1H-detected MAS NMR and 13C DNP NMR – practical aspects
Source: J Biomol NMR. 2024 Jun 21;78(3):179–92. doi: 10.1007/s10858-024-00444-9 (PMC7616530; doi:10.1007/s10858-024-00444-9)
Supplement: Supplementary file 1 — Supplementary Material 1 [file 10858_2024_444_MOESM1_ESM.pdf]

Supporting Information

Sedimentation of large, soluble proteins up to 140 kDa for ^{1}H-detected MAS NMR and ^{13}C DNP NMR – practical aspects

Dallas Bell[1,2], Florian Lindemann[2], Lisa Gerland[2], Hanna Aucharova[3], Alexander Klein[3], Daniel Friedrich[4], Matthias Hiller[2], Kristof Grohe[5], Tobias Meier[5], Barth van Rossum[2], Anne Diehl[2], Jon Hughes[6,7], Leonard J. Mueller[8], Rasmus Linser[3], Anne-Frances Miller[1,9] and Hartmut Oschkinat[2]

Affiliations

[1]Faculty II-Mathematics and Natural Sciences, Technische Universität Berlin, Straße des 17. Juni 135, 10623 Berlin, Germany

[2]Leibniz-Forschungsinstitut für Molekulare Pharmakologie, Robert-Rössle-Str. 10, 13125 Berlin, Germany

[3]Department of Chemistry and Chemical Biology, TU Dortmund University, Otto-Hahn-Str. 4a, 44227 Dortmund, Germany.

[4]Department of Chemistry and Biochemistry, University of Cologne, Greinstr. 4, 50939 Cologne, Germany

[5]Bruker BioSpin GmbH & Co. KG, Rudolf-Plank-Str. 23, 76275 Ettlingen, Germany

[6]Justus Liebig University, Institute for Plant Physiology, Senckenbergstr. 3, 35360 Gießen, Germany

[7]Free University of Berlin, Department of Physics, Arnimallee 14, 14195 Berlin, Germany

[8]Department of Chemistry, University of California - Riverside, CA 92521 Riverside, USA

[9]Department of Chemistry, University of Kentucky, KY 40506 Lexington, USA

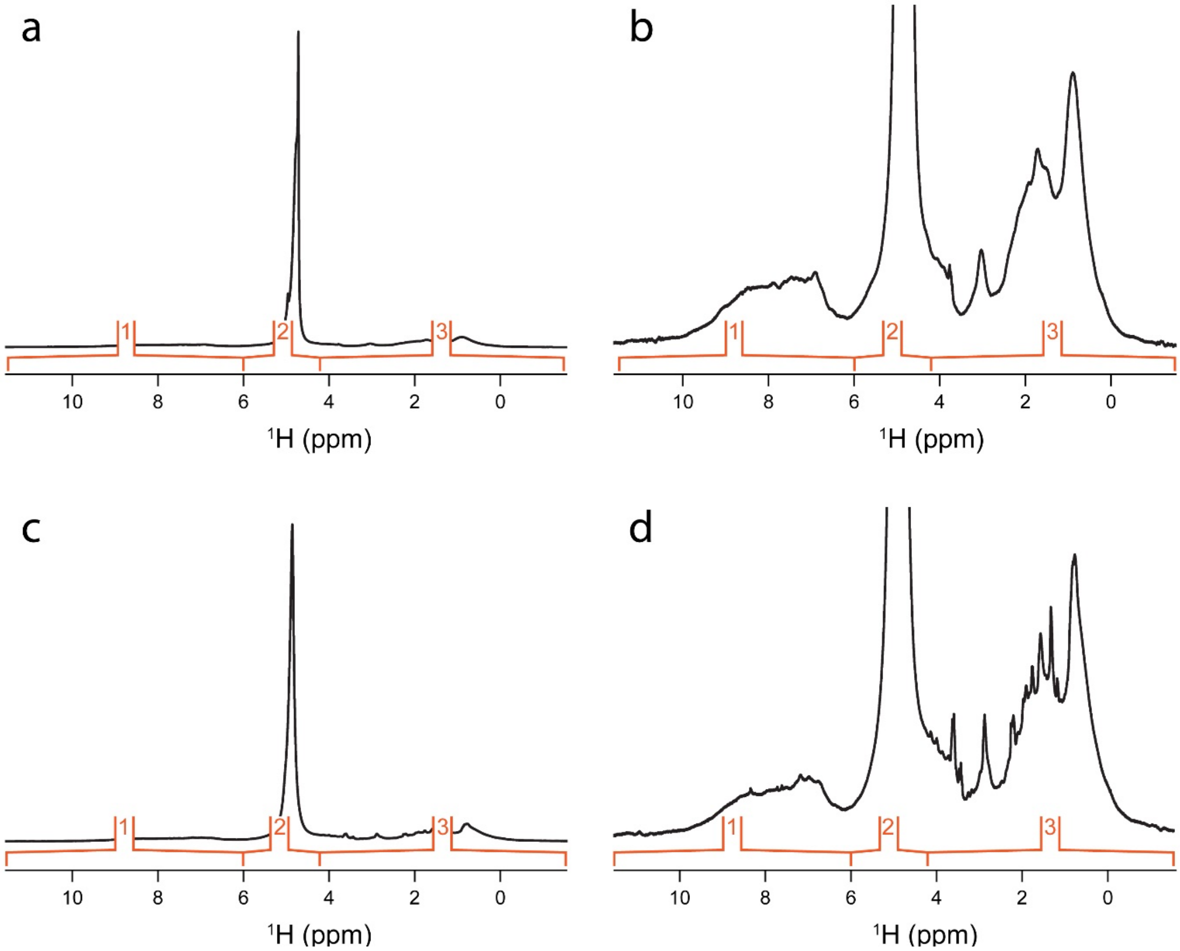

Fig. S1 1D ^{1}H spectra used to determine the water content and its change over time. A 0.7 mm diameter rotor containing sedimented *Sa*ETF was measured at 900 MHz ^{1}H Larmor frequency and at 100 kHz MAS. Spectra and integration regions of freshly filled sample (a and b) and after 1 year (c and d) are shown. The determined integrals are: (a and b) |1|: 1.00; |2|: 12.35; |3|: 2.24, (c and d) |1|: 1.00; |2|: 13.77; |3|: 2.86. The water content was roughly estimated as follows: The integral of the region between 4.2 and 6 ppm was considered to reflect largely water, and the areas to the right and left protons of the protein. Protein signals under the water lead to an error, yet the water line had a hump extending towards 3 ppm, leading to an error in the opposite direction. We assumed a compensation of both. For the fresh sample, the integral of the water area is 3.8 times larger than the sum of the other two that are reflecting the protein. Since we are interested in the water/protein ratio, it is fair to assume that the protein is represented by integrals 1 and 3 that reflect 4862 protons (for one molecule), and the water signal contains 2 protons times x molecules. With an integral ratio of 3.8 and molecular weights of 66843 g/mol (protein) and 18 g/mol (water) a 2.5-fold excess of water (w/w) is obtained for the fresh sample.

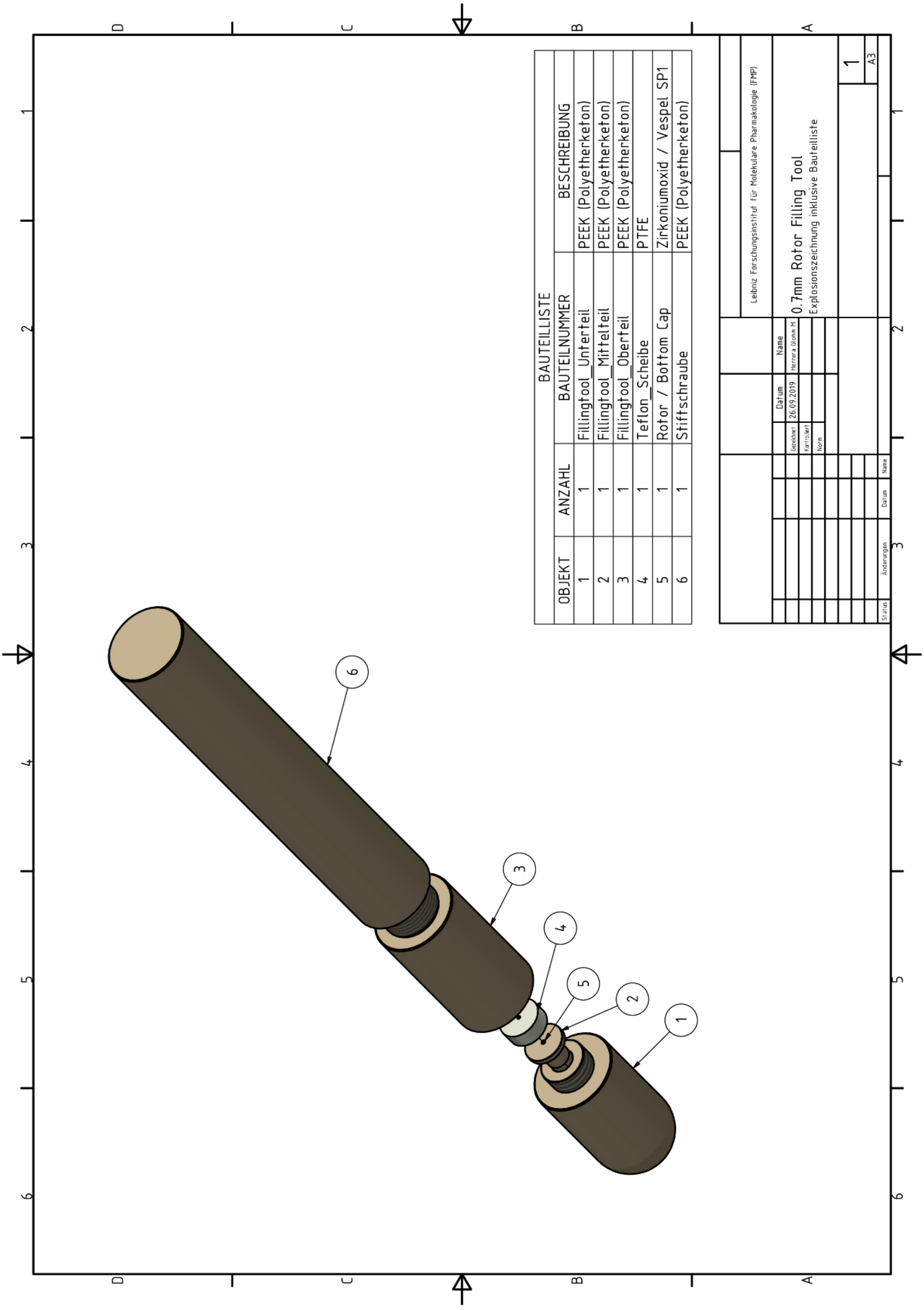

Fig. S2 Technical drawing of the 0.7mm filling tool, overview.

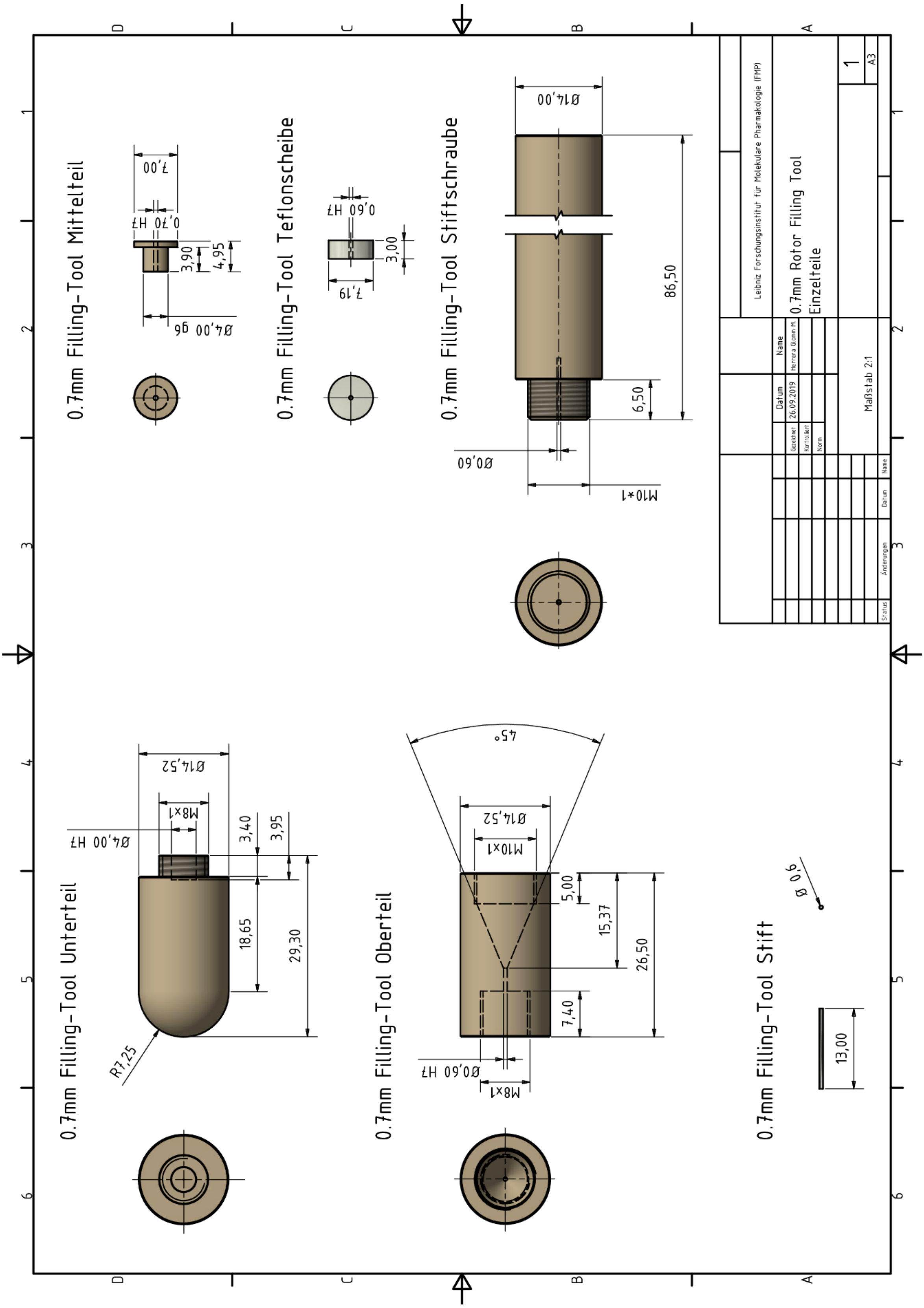

Fig. S3 Technical drawings of the 0.7mm filling tool, detailed view.

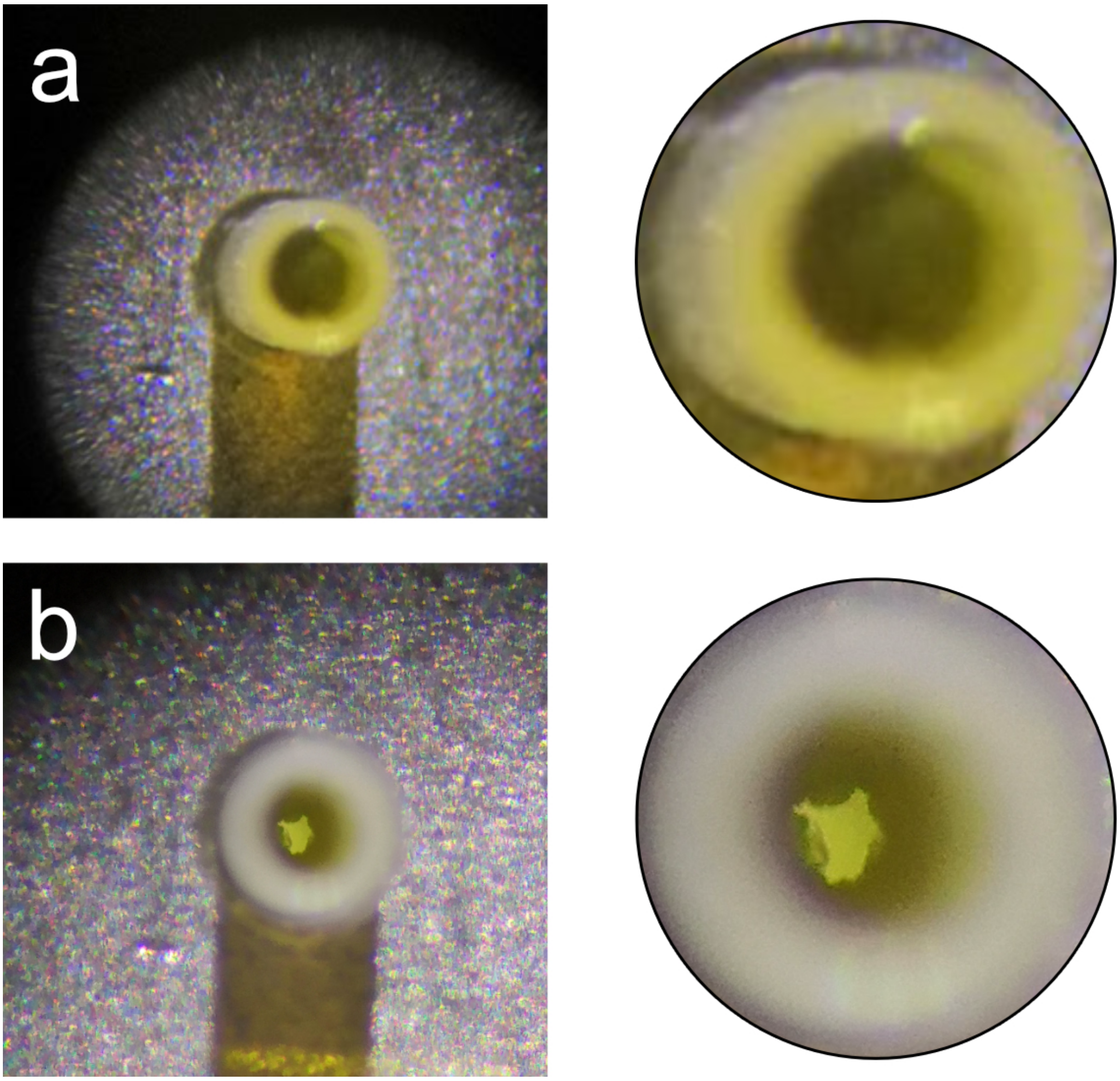

Fig. S4 Effect of magic angle spinning on the sample inside the NMR rotor. (a) Top view of a 0.7 mm diameter rotor directly after filling *St*TS by ultracentrifugation. Due to the filling procedure, the bottom cap is attached. (b) Top view of the identical 0.7 mm rotor after multiple sessions of MAS NMR measurements at 100 kHz spinning after removing both caps. Enlarged images are shown on the right.

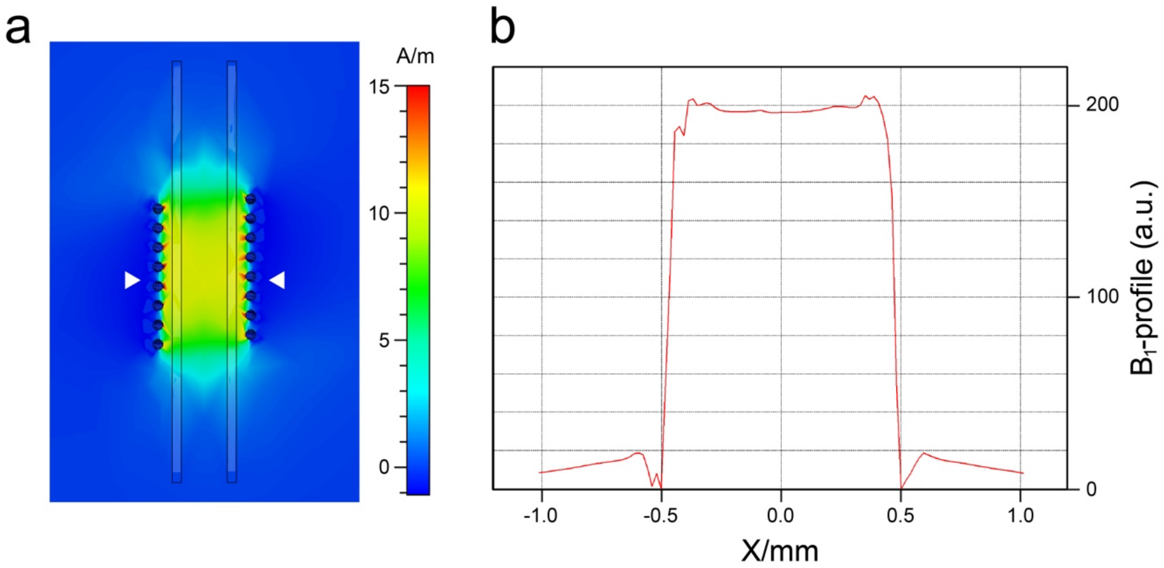

Fig. S5 (a) B1 profile of a 0.7mm rotor. (b) profile perpendicular to the rotor axis, taken in the center (see white arrows in (a)).

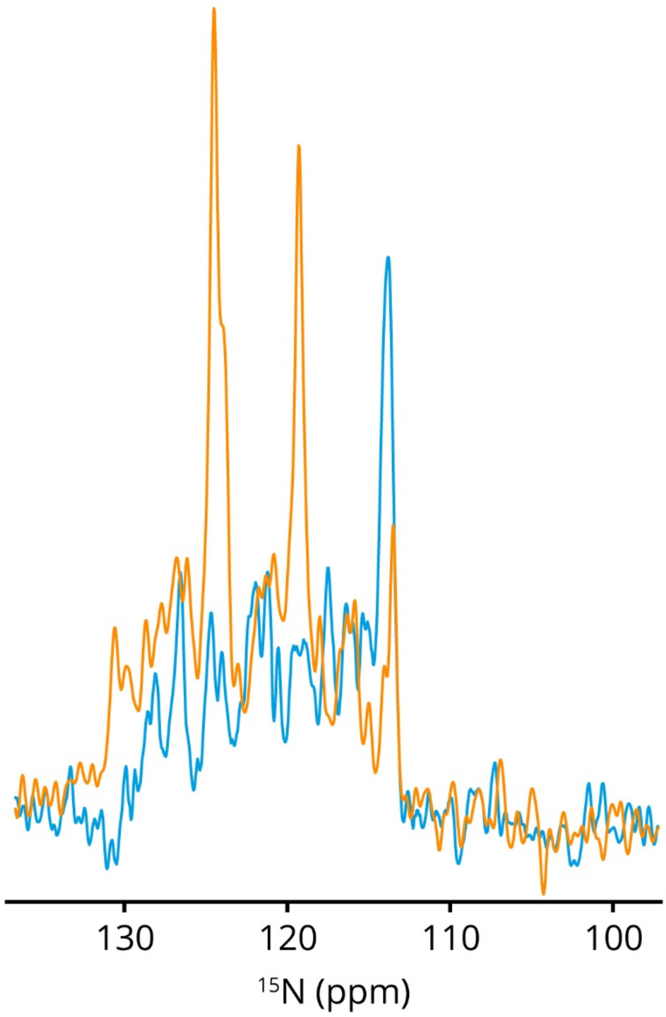

Fig. S6 1D slices along a ^{1}H chemical shift of 8.95 ppm from ^{1}H-^{15}N correlations of deuterated *Sa*ETF by solid-state NMR (CP-based, yellow) and solution NMR (INEPT-based, blue). Full spectra are shown in main text Fig. 3.

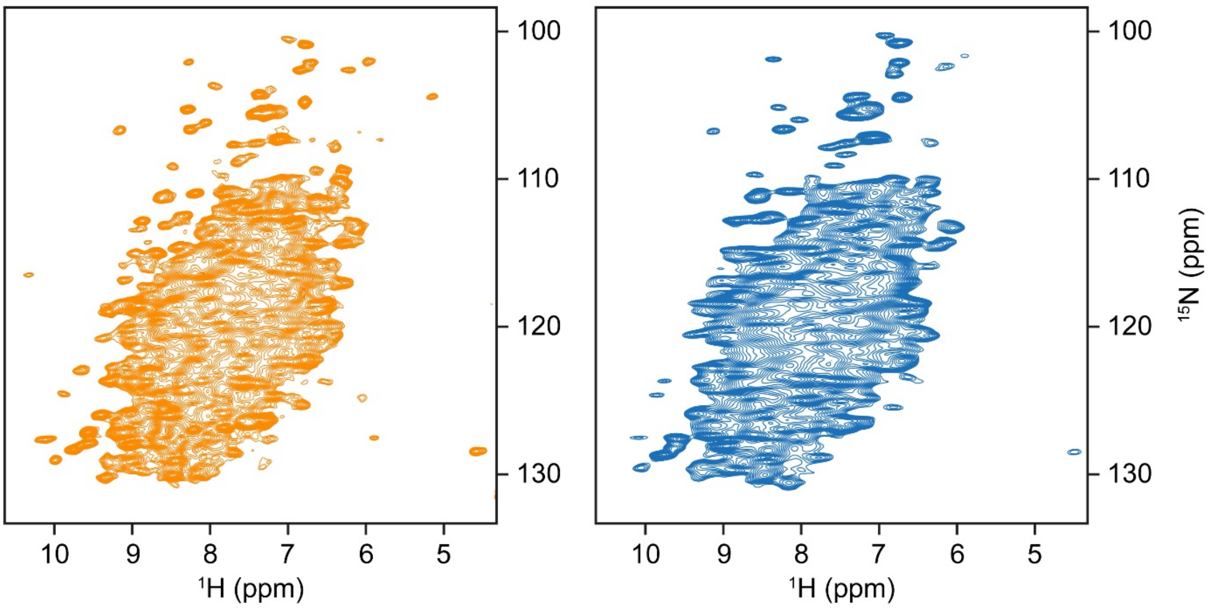

Fig. S7 Comparison of CP-based ^{1}H-^{15}N *Sa*ETF spectra to examine the change of the sample over time. Measurements were conducted with a 0.7 mm rotor spinning at 100 kHz MAS on a spectrometer with 900 MHz ^{1}H Larmor frequency directly after filling (orange, from the main text) and after one year of storage (blue).

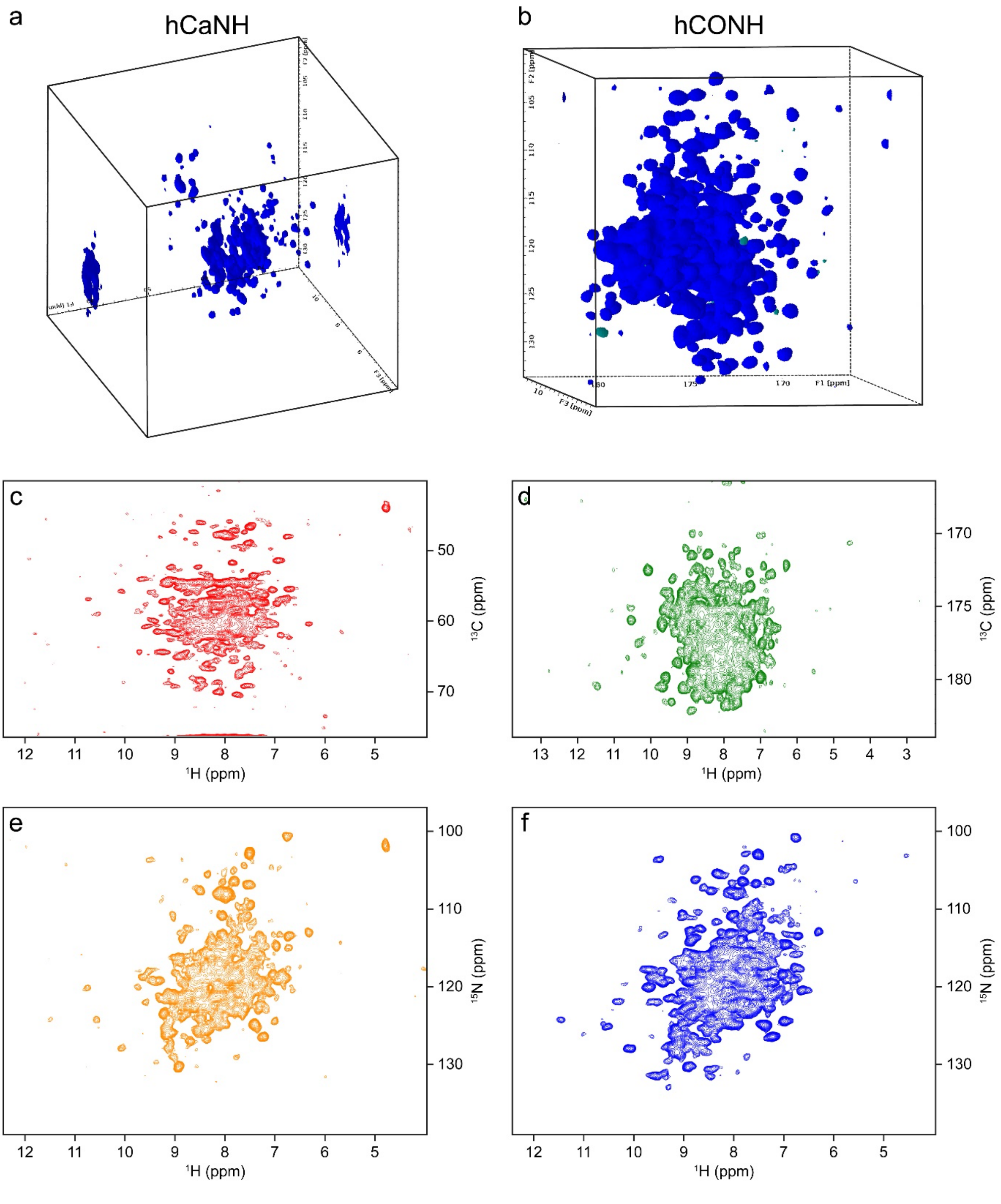

Fig. S8 3D hCANH and hCONH spectra acquired on ^{1}H,^{13}C,^{15}N-labelled *St*TS, filled into a 0.7 mm rotor and at 900 MHz ^{1}H Larmor frequency and 100 kHz MAS. (a) Cube representation of the hCANH. (b) Cube representation of the hCONH. (c) ^{1}H-^{13}C projection of the hCANH. (d) ^{1}H-^{13}C projection of the hCONH. (e) ^{1}H-^{15}N projection of the hCANH. (f) ^{1}H-^{15}N projection of the hCONH.

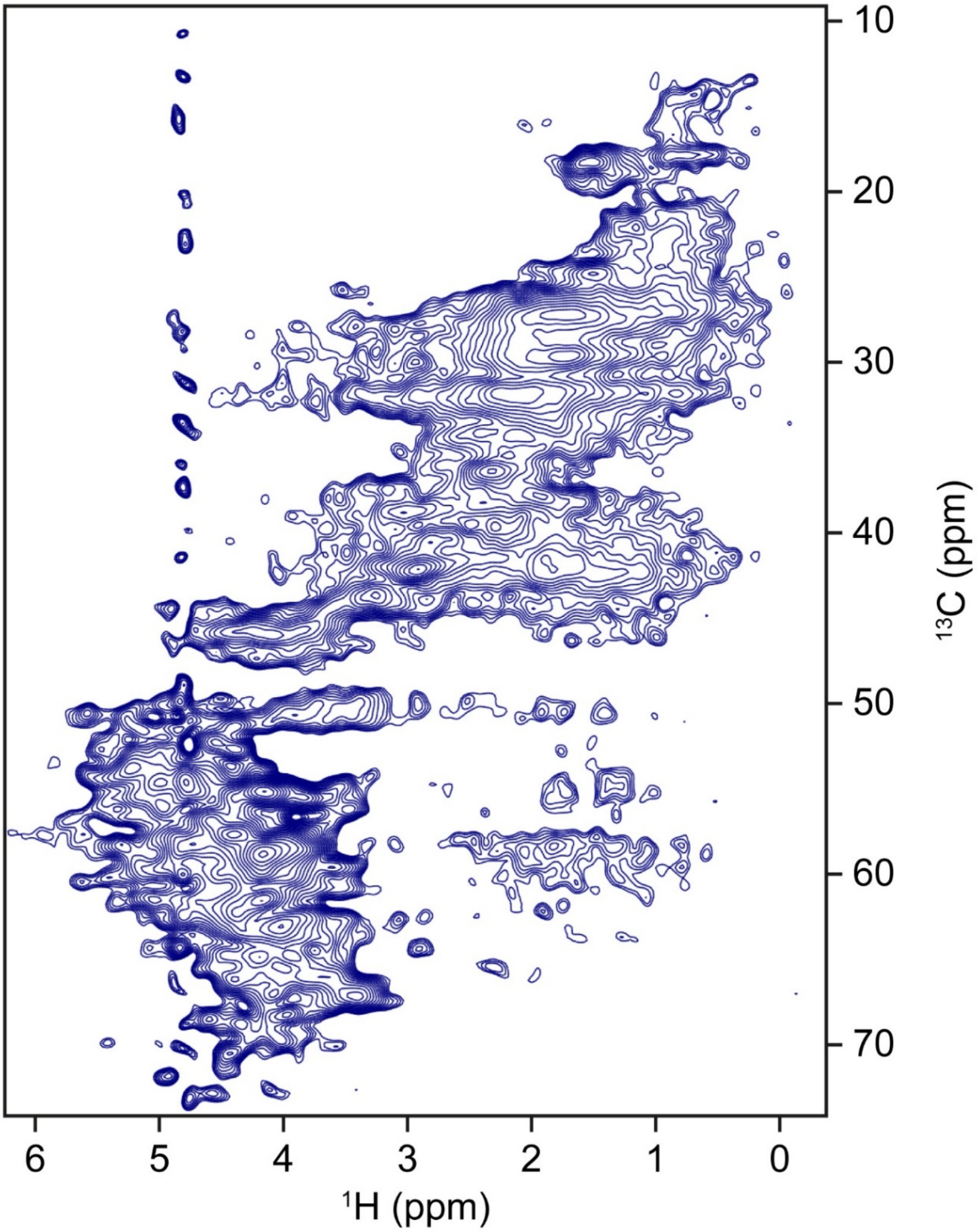

Fig. S9 CP-based ^{1}H-^{13}C correlation of *St*TS, recorded with a 0.7 mm rotor at 100 kHz MAS and 900 MHz ^{1}H Larmor frequency.

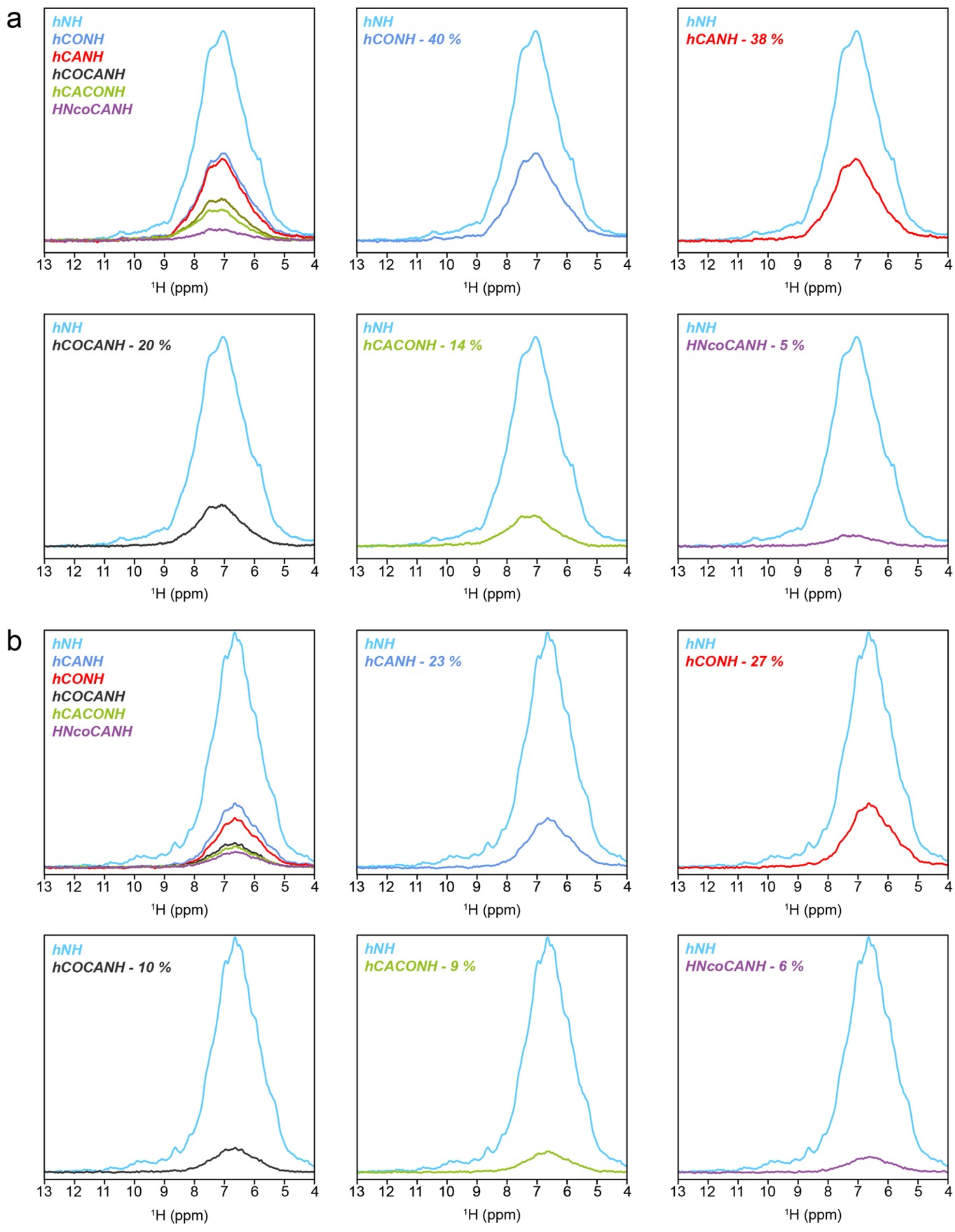

Fig. S10 Comparisons of transfer efficiencies on *Pf*TrpB at 700 MHz ^{1}H Larmor frequency. Individual bulk intensities were determined on the first 1D free induction decay with 256 scans and compared to the bulk intensity of the first 1D experiment of a 2D hNH experiment (also measured with 256 scans). The relative intensities are given as percentages. (a) Measurements using iFD-labelled protein in a 0.7 mm rotor at 100 kHz. The ^{1}H bulk T_1 time was determined to be 750 ms, a recycle delay of 1 s was used. (b) Measurements using ^{2}H, ^{13}C, ^{15}N-labelled and back-exchanged sedimented into a 1.3 mm rotor at 55 kHz MAS. The ^{1}H bulk T_1 time was determined to be 800 ms, a recycle delay of 1 s was used.

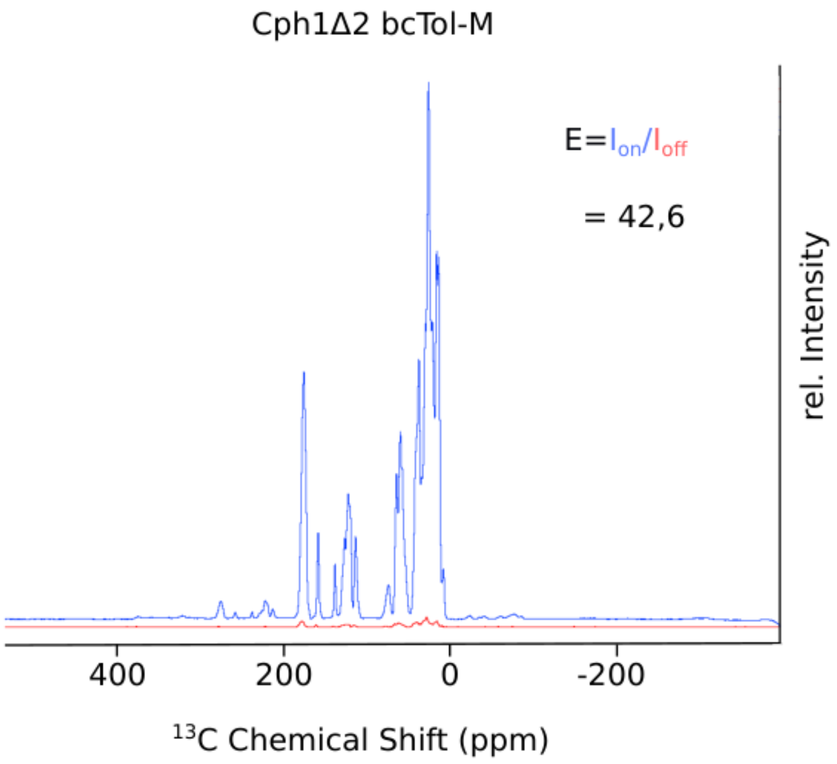

Fig. S11 DNP enhancement obtained by the radical BcTol-M on Cph1Δ2 from *Synechocystis* at 800 MHz ^{1}H Larmor frequency spinning at 20 kHz MAS. The spectra shown were acquired with (blue) and without (red) microwave irradiation.

Table S1 Acquisition parameters for the 2D ^{1}H-^{15}N spectra of *Sa*ETF.

**Acquisition parameters**	CP-based hNH (Figs. 2 and 3)		Solution ^{1}H-^{15}N HSQC (Fig. 3)	
	F2	F1	F2	F1
Quad. Mode		States-TPPI		States-TPPI
TD points	1024	640	1024	512
Nucleus	^{1}H	^{15}N	^{1}H	^{15}N
SW / ppm	39.7	99.7	16.3	79.5
Aq.time / ms	14.3	35.2	34.8	35.3
Scans	32		64	

**Acquisition parameters**	CP-based hNH @ 289 K (Fig. 4)		CP-based hNH @ 313 K (Fig. 4)	
	F2	F1	F2	F1
Quad. Mode		States-TPPI		States-TPPI
TD points	2048	512	2048	512
Nucleus	^{1}H	^{15}N	^{1}H	^{15}N
SW / ppm	39.7	80.0	39.7	85.7
Aq.time / ms	28.7	35.1	34.8	32.8
Scans	32		32	

**Acquisition parameters**	INEPT-based hNH @ 289 K (Fig. 4)		INEPT-based hNH @ 313 K (Fig. 4)	
	F2	F1	F2	F1
Quad. Mode		States-TPPI		States-TPPI
TD points	2048	384	2048	256
Nucleus	^{1}H	^{15}N	^{1}H	^{15}N
SW / ppm	39.7	80.0	39.7	85.7
Aq.time / ms	28.7	26.3	28.7	16.4
Scans	32		48	

**Acquisition parameters**		
	CP-based hNH after 1 year (Fig. S6)	
	F2	F1
Quad. Mode		States-TPPI
TD points	2048	2048
Nucleus	^{1}H	^{15}N
SW / ppm	39.7	142.0
Aq.time / ms	28.7	79.1
Scans	32	

Table S2 RF fields used to obtain the 2D ^{1}H-^{15}N spectra of *Sa*ETF.

CP-based hNH (Figs. 2 and 3)			Solution ^{1}H-^{15}N HSQC (Fig. 3)	
**Hard pulses**	μs	RF / kHz	μs	RF / kHz
^{1}H	0.85	294.12	10.29	24.30
^{15}N	5	50	40	6.25
^{13}C	5	50		

CP-based hNH @ 289 K (Fig. 4)			CP-based hNH @ 313 K (Fig. 4)	
**Hard pulses**	μs	RF / kHz	μs	RF / kHz
^{1}H	0.9	277.78	1	250.0
^{15}N	4.6	54.35	5	50
^{13}C	2.5	100	5	50

INEPT-based hNH @ 289 K (Fig. 4)			INEPT-based hNH @ 313 K (Fig. 4)	
**Hard pulses**	μs	RF / kHz	μs	RF / kHz
^{1}H	0.9	277.78	1	250.0
^{15}N	4.6	54.35	5	50
^{13}C	5	50	5	50

CP-based hNH after 1 year (Fig. S6)		
**Hard pulses**	μs	RF / kHz
^{1}H	0.9	277.78
^{15}N	5.7	44.17
^{13}C	2.5	100

Table S3 CP conditions used to obtain the hNH spectra of *Sa*ETF.

**hNH** (Figs. 2 and 3)							
H-N CP		RF ^{1}H / kHz	RF ^{15}N / kHz	N-H CP		RF ^{1}H / kHz	RF ^{15}N / kHz
Contact time/μs	800	68.6	34.13	Contact time/μs	600	68.6	29.22
Shape		rectangular	100-60 tang.	Shape		rectangular	60-100 tang.

**hNH @ 289 K** (Fig. 4)							
H-N CP		RF ^{1}H / kHz	RF ^{15}N / kHz	N-H CP		RF ^{1}H / kHz	RF ^{15}N / kHz
Contact time/μs	1000	78.16	37.23	Contact time/μs	700	78.16	33.30
Shape		rectangular	100-60 tang.	Shape		rectangular	60-100 tang.

**hNH @ 313 K** (Fig. 4)							
H-N CP		RF ^{1}H / kHz	RF ^{15}N / kHz	N-H CP		RF ^{1}H / kHz	RF ^{15}N / kHz
Contact time/μs	800	70.84	36.40	Contact time/μs	400	70.84	30.06
Shape		rectangular	100-60 tang.	Shape		rectangular	60-100 tang.

**hNH after 1 year** (Fig. S6)							
H-N CP		RF ^{1}H / kHz	RF ^{15}N / kHz	N-H CP		RF ^{1}H / kHz	RF ^{15}N / kHz
Contact time/μs	1100	78.16	31.97	Contact time/μs	700	78.16	28.92
Shape		rectangular	100-60 tang.	Shape		rectangular	60-100 tang.

Table S4 Acquisition parameters for the spectra of *St*TS.

**Acquisition parameters**	hNH (Fig. 5a)		hNH (Fig. 5b)	
	F2	F1	F2	F1
Quad. Mode		States-TPPI		States-TPPI
TD points	2048	400	2048	400
Nucleus	^{1}H	^{15}N	^{1}H	^{15}N
SW / ppm	39.7	137	39.7	78.34
Aq.time / ms	28.7	16	28.7	28
Scans	128		128	

**Acquisition parameters**	hCANH (Fig. S7a, c and e)		
	F3	F2	F1
Quad. Mode		States-TPPI	States-TPPI
TD points	1024	64	126
Nucleus	^{1}H	^{15}N	^{13}C
SW / ppm	39.7	34.3	44.2
Aq.time / ms	14.3	10.2	6.4
Scans	16		

**Acquisition parameters**	hCONH (Fig. S7b, d and f)		
	F3	F2	F1
Quad. Mode		States-TPPI	States-TPPI
TD points	1024	80	64
Nucleus	^{1}H	^{15}N	^{13}C
SW / ppm	39.7	34.3	17.7
Aq.time / ms	14.3	12.8	8.0
Scans	24		

Table S5 RF fields and CP conditions used for the spectra of *St*TS.

	hNH (Fig. 5a and b)		hCANH and hCONH (Fig. S7)	
**Hard pulses**	µs	RF / kHz	µs	RF / kHz
^{1}H	0.85	294.11	2.5	100
^{15}N	5	50	5	50
^{13}C	5	50	5	50

**hNH** (Fig. 5a and b)							
H-N CP		RF ^{1}H / kHz	RF ^{15}N / kHz	N-H CP		RF ^{1}H / kHz	RF ^{15}N / kHz
Contact time/µs	1200	70.15	34.61	Contact time/µs	800	70.15	28.87
Shape		rectangular	100-60 tang.	Shape		rectangular	60-100 tang.

**hCANH** (Fig. S7a, c and e)				
H-C CP		RF ^{1}H / kHz	RF ^{15}N / kHz	RF ^{13}C / kHz
Contact time/µs	1400	71.98		23.61
Shape		80-100 ramp		rectangular
CA-N				
Contact time/µs	10000		23.71	71.39
Shape			rectangular	70-100 tang.
H-N CP		RF ^{1}H / kHz	RF ^{15}N / kHz	RF ^{13}C / kHz
Contact time/µs	800	70.89	28.2	
Shape		80-100 ramp	rectangular	

**hCONH** (Fig. S7b, d and f)				
H-C CP		RF ^{1}H / kHz	RF ^{15}N / kHz	RF ^{13}C / kHz
Contact time/µs	1400	71.98		23.0
Shape		80-100 ramp		rectangular
CO-N CP		RF ^{1}H / kHz	RF ^{15}N / kHz	RF ^{13}C / kHz
Contact time/µs	10000		25.52	67.94
Shape			rectangular	70-100 tang
H-N CP		RF ^{1}H / kHz	RF ^{15}N / kHz	RF ^{13}C / kHz
Contact time/µs	800	70.89	21.65	
Shape		80-100 ramp	rectangular	

Table S6 Acquisition parameters for the 2D hNH spectrum of the u-[^{13}C,^{15}N] labeled sample of the 2B9 mutant of *Pf*TrpB spun at 100 kHz MAS in a 0.7 mm rotor as shown in main text Fig. 6.

**Acquisition parameters**		
	F2	F1
Quad. Mode		States-TPPI
TD points	800	138
Nucleus	^{1}H	^{15}N
SW / ppm	39.7	39.1
Aq.time / ms	14.4	24.8
Scans	128	

Table S7 RF fields and CP conditions used to obtain the ^{1}HN bulk intensities in main text Fig. 6 and Fig. S9 for the u-[^{13}C,^{15}N] labeled sample of the 2B9 mutant of *Pf*TrpB spun at 100 kHz MAS in a 0.7 mm rotor.

**Hard pulses**	µs	RF / kHz
^{1}H	1.4	178.57
^{15}N	2.8	89.29
^{13}C	2.6	96.15

**hNH**				
H-N CP		RF ^{1}H / kHz	RF ^{15}N / kHz	RF ^{13}C / kHz
Contact time/µs	1150	80.4	28.2	
Shape		100-50 tang.	rectangular	

**hCANH**				
H-C CP		RF ^{1}H / kHz	RF ^{15}N / kHz	RF ^{13}C / kHz
Contact time/µs	300	80.4		31.3
Shape		100-50 tang.		rectangular
Ca-N CP		RF 1H / kHz	RF 15N / kHz	RF 13C / kHz
Contact time/µs	11000		66.6	31.7
Shape			90-100 ramp	rectangular
H-N CP		RF ^{1}H / kHz	RF ^{15}N / kHz	RF ^{13}C / kHz
Contact time/µs	500	80.4	28.2	
Shape		100-50 tang.	rectangular	

**hCONH**				
H-C CP		RF ^1H / kHz	RF ^{15}N / kHz	RF ^{13}C / kHz
Contact time/µs	1500	80.4		29.5
Shape		100-50 tang.		rectangular
CO-N CP		RF ^1H / kHz	RF ^{15}N / kHz	RF ^{13}C / kHz
Contact time/µs	11000		66.6	31.3
Shape			90-100 ramp	rectangular
H-N CP		RF ^1H / kHz	RF ^{15}N / kHz	RF ^{13}C / kHz
Contact time/µs	500	80.4	28.2	
Shape		100-50 tang.	rectangular	

**hCACONH**				
H-C CP		RF ^1H / kHz	RF ^{15}N / kHz	RF ^{13}C / kHz
Contact time/µs	650	80.4		31.3
Shape		100-50 tang.		rectangular
CO-CA BSH-CP		RF ^1H / kHz	RF ^{15}N / kHz	RF ^{13}C / kHz
Trim pulse/µs	1.04			96.2
Contact time/µs	5500			42.2
Shape				90-100 ramp
CO-N CP		RF ^1H / kHz	RF ^{15}N / kHz	RF ^{13}C / kHz
Contact time/µs	11000		66.6	31.3
Shape			90-100 ramp	rectangular
H-N CP		RF ^1H / kHz	RF ^{15}N / kHz	RF ^{13}C / kHz
Contact time/µs	500	80.4	28.2	
Shape		100-50 tang.	rectangular	

**hCOCANH**				
H-C CP		RF ^{1}H / kHz	RF ^{15}N / kHz	RF ^{13}C / kHz
Contact time/µs	1500	80.4		29.5
Shape		100-50 tang.		rectangular
CO-CA BSH-CP		RF ^{1}H / kHz	RF ^{15}N / kHz	RF ^{13}C / kHz
Trim pulse/µs	1.04			96.2
Contact time/µs	5500			44.7
Shape				90-100 ramp
Ca-N CP		RF ^{1}H / kHz	RF ^{15}N / kHz	RF ^{13}C / kHz
Contact time/µs	11000		66.6	31.7
Shape			90-100 ramp	rectangular
H-N CP		RF ^{1}H / kHz	RF ^{15}N / kHz	RF ^{13}C / kHz
Contact time/µs	500	80.4	28.2	
Shape		100-50 tang.	rectangular	

**HNcoCANH**				
H-N CP		RF ^{1}H / kHz	RF ^{15}N / kHz	RF ^{13}C / kHz
Contact time/µs	1500	80.4	28.2	
Shape		100-50 tang.	rectangular	
CO-N CP		RF ^{1}H / kHz	RF ^{15}N / kHz	RF ^{13}C / kHz
Contact time/µs	11000		66.6	31.3
Shape			90-100 ramp	rectangular
CO-CA BSH-CP		RF ^{1}H / kHz	RF ^{15}N / kHz	RF ^{13}C / kHz
Trim pulse/µs	1.04			96.2
Contact time/µs	5500			44.7
Shape				90-100 ramp
Ca-N CP		RF ^{1}H / kHz	RF ^{15}N / kHz	RF ^{13}C / kHz
Contact time/µs	11000		66.6	31.7
Shape			90-100 ramp	rectangular
H-N CP		RF ^{1}H / kHz	RF ^{15}N / kHz	RF ^{13}C / kHz
Contact time/µs	500	80.4	28.2	
Shape		100-50 tang.	rectangular	

Table S8 Acquisition parameters for the 2D hNH spectrum of the u-[^{2}H,^{13}C,^{15}N] labeled sample of the 2B9 mutant of *Pf*TrpB spun at 55 kHz MAS in a 1.3 mm rotor as shown in main text Fig. 6.

**Acquisition parameters**		
	F2	F1
Quad. Mode		States-TPPI
TD points	800	138
Nucleus	^{1}H	^{15}N
SW / ppm	39.7	39.1
Aq.time / ms	19.2	24.8
Scans	128	

Table S9 RF fields and CP conditions used to obtain the ^{1}HN bulk intensities in main text Fig. 6 and Fig. S9 for the u-[^{2}H,^{13}C,^{15}N] labeled sample of the 2B9 mutant of *Pf*TrpB spun at 55 kHz MAS in a 1.3 mm rotor.

**Hard pulses**	µs	RF / kHz
^{1}H	1.6	156.25
^{15}N	5	50.00
^{13}C	3.2	78.13

**hNH**				
H-N CP		RF ^{1}H / kHz	RF ^{15}N / kHz	RF ^{13}C / kHz
Contact time/µs	150	13.4	42.5	
Shape		100-50 tang.	rectangular	

**hCANH**				
H-C CP		RF ^{1}H / kHz	RF ^{15}N / kHz	RF ^{13}C / kHz
Contact time/µs	1200	15.9		45.4
Shape		100-80 ramp		rectangular
Ca-N CP		RF 1H / kHz	RF 15N / kHz	RF 13C / kHz
Contact time/µs	11000		38.3	18.7
Shape			90-100 ramp	rectangular
H-N CP		RF ^{1}H / kHz	RF ^{15}N / kHz	RF ^{13}C / kHz
Contact time/µs	150	13.4	42.5	
Shape		100-50 tang.	rectangular	

**hCONH**				
H-C CP		RF ^{1}H / kHz	RF ^{15}N / kHz	RF ^{13}C / kHz
Contact time/µs	1300	15.9		47.0
Shape		100-80 ramp		rectangular
CO-N CP		RF ^{1}H / kHz	RF ^{15}N / kHz	RF ^{13}C / kHz
Contact time/µs	11000		38.3	18.7
Shape			90-100 ramp	rectangular
H-N CP		RF ^{1}H / kHz	RF ^{15}N / kHz	RF ^{13}C / kHz
Contact time/µs	150	13.4	42.5	
Shape		100-50 tang.	rectangular	

**hCACONH**				
H-C CP		RF ^{1}H / kHz	RF ^{15}N / kHz	RF ^{13}C / kHz
Contact time/µs	1300	15.9		46.0
Shape		100-80 ramp		rectangular
CO-CA BSHCP		RF ^{1}H / kHz	RF ^{15}N / kHz	RF ^{13}C / kHz
Trim pulse/µs	1.45			78.13
Contact time/µs	6500			28.5
Shape				90-100 ramp
Ca-N CP		RF ^{1}H / kHz	RF ^{15}N / kHz	RF ^{13}C / kHz
Contact time/µs	11000		38.3	18.7
Shape			90-100 ramp	rectangular
H-N CP		RF ^{1}H / kHz	RF ^{15}N / kHz	RF ^{13}C / kHz
Contact time/µs	150	13.4	42.5	
Shape		100-50 tang.	rectangular	

**hCOCANH**				
H-C CP		RF ^{1}H / kHz	RF ^{15}N / kHz	RF ^{13}C / kHz
Contact time/µs	1300	15.9		47.0
Shape		100-80 ramp		rectangular
CO-CA BSH-CP		RF ^{1}H / kHz	RF ^{15}N / kHz	RF ^{13}C / kHz
Trim pulse/µs	1.45			78.13
Contact time/µs	6500			28.5
Shape				90-100 ramp
Ca-N CP		RF ^{1}H / kHz	RF ^{15}N / kHz	RF ^{13}C / kHz
Contact time/µs	11000		38.3	18.7
Shape			90-100 ramp	rectangular
H-N CP		RF ^{1}H / kHz	RF ^{15}N / kHz	RF ^{13}C / kHz
Contact time/µs	150	13.4	42.5	
Shape		100-50 tang.	rectangular	

**HNcoCANH**				
H-N CP		RF ^{1}H / kHz	RF ^{15}N / kHz	RF ^{13}C / kHz
Contact time/µs	150	13.4	42.5	
Shape		100-50 tang.	rectangular	
CO-N CP		RF ^{1}H / kHz	RF ^{15}N / kHz	RF ^{13}C / kHz
Contact time/µs	11000		38.3	18.7
Shape			90-100 ramp	rectangular
CO-CA BSHCP		RF ^{1}H / kHz	RF ^{15}N / kHz	RF ^{13}C / kHz
Trim pulse/µs	1.45			78.13
Contact time/µs	6500			28.5
Shape				90-100 ramp
Ca-N CP		RF ^{1}H / kHz	RF ^{15}N / kHz	RF ^{13}C / kHz
Contact time/µs	11000		38.3	18.7
Shape			90-100 ramp	rectangular
H-N CP		RF ^{1}H / kHz	RF ^{15}N / kHz	RF ^{13}C / kHz
Contact time/µs	500	13.4	42.5	
Shape		100-50 tang.	rectangular	
